# Neurologic and Psychological Outcomes 2 Years After Multisystem Inflammatory Syndrome in Children

**DOI:** 10.1001/jamanetworkopen.2025.12487

**Published:** 2025-06-02

**Authors:** Caitlin K. Rollins, David Wypij, Laura D. Zambrano, Johanna Calderon, Alex M. Taylor, Jennifer Worhach, Susan Rodriguez, Paul A. Licht, Moshe Maiman, Nicholas Hart, Mary Beth F. Son, Joe Kossowsky, Matthew L. Friedman, Charlotte V. Hobbs, Michele Kong, Aline B. Maddux, Jennifer L. McGuire, Mary Allen Staat, Lael M. Yonker, Maitreyi Mazumdar, Jane W. Newburger, Adrienne G. Randolph, Angela P. Campbell

**Affiliations:** 1 Department of Neurology, Boston Children’s Hospital, Boston, Massachusetts; 2 Department of Neurology, Harvard Medical School, Boston, Massachusetts; 3 Department of Biostatistics, Harvard T.H. Chan School of Public Health, Boston, Massachusetts; 4 Department of Cardiology, Boston Children’s Hospital, Boston, Massachusetts; 5 Department of Pediatrics, Harvard Medical School, Boston, Massachusetts; 6 Coronavirus and Other Respiratory Viruses Division, National Center for Immunization and Respiratory Diseases, Centers for Disease Control and Prevention, Atlanta, Georgia; 7 National Institute of Health and Medical Research INSERM U1046, PhyMedExp, Montpellier, France; 8 Department of Psychiatry and Behavioral Sciences, Boston Children’s Hospital, Boston, Massachusetts; 9 Department of Psychiatry, Harvard Medical School, Boston, Massachusetts; 10 Department of Anesthesiology, Critical Care, and Pain Medicine, Boston Children’s Hospital, Boston, Massachusetts; 11 Division of Immunology, Department of Pediatrics, Boston Children’s Hospital, Boston, Massachusetts; 12 Division of Pediatric Critical Care Medicine, Department of Pediatrics, Indiana University School of Medicine and Riley Hospital for Children, Indianapolis; 13 Division of Infectious Diseases, Department of Pediatrics, University of Mississippi Medical Center, Jackson; 14 Division of Pediatric Critical Care Medicine, Department of Pediatrics, University of Alabama at Birmingham; 15 Department of Pediatrics, Section of Critical Care Medicine, University of Colorado School of Medicine and Children’s Hospital Colorado, Aurora; 16 Division of Neurology, The Children’s Hospital of Philadelphia, Philadelphia, Pennsylvania; 17 Department of Neurology, Perelman School of Medicine, University of Pennsylvania, Philadelphia; 18 Division of Infectious Diseases, Department of Pediatrics, University of Cincinnati, Cincinnati Children’s Hospital Medical Center, Cincinnati, Ohio; 19 Division of Pediatric Pulmonary and Mucosal Immunology and Biology Research Center, Department of Pediatrics, Massachusetts General Hospital, Boston; 20 Department of Anaesthesia, Harvard Medical School, Boston, Massachusetts; 21 Department of Microbiology, University of Mississippi Medical Center, Jackson; 22 Department of Pediatrics, Perelman School of Medicine, University of Pennsylvania, Philadelphia; 23 Division of Pulmonology, Massachusetts General Hospital, Boston

## Abstract

**Importance:**

Neurologic and psychological sequelae are observed 1 year after hospitalization for multisystem inflammatory syndrome in children (MIS-C), but whether these concerns persist is not known.

**Objective:**

To examine the trajectory of neurologic, psychological, and quality-of-life sequelae up to 2 years after MIS-C.

**Design, Setting, and Participants:**

This longitudinal cohort study assessed children diagnosed with MIS-C from August 1, 2020, to August 31, 2021, and matched sibling and community controls, when available. The study was conducted 6 to 12 months and 18 to 24 months after discharge from a US or Canadian hospital. Data analysis was performed from May 2024 to January 2025.

**Exposure:**

Hospitalization for MIS-C.

**Main Outcomes and Measures:**

A central study site remotely administered a structured interview, surveys, neuropsychological assessment, and neurologic examination. Group differences were assessed using generalized estimating equations, accounting for matching. Variables extracted from hospital records included intensive care unit admission and echocardiographic left ventricular ejection fraction (LVEF).

**Results:**

Overall, 95 participants were included in the study; 93 of 108 participants (86%) returned from the year 1 study and 2 participants were added in year 2 (median [IQR] age, 12.6 [11.0-15.7] years; 38 [40%] female and 57 [60%] male). Fifty-nine patients with MIS-C (mean [SD] age, 13.2 [4.0] years; 39 [66%] male) and 36 controls (mean [SD] age, 13.5 [3.5] years; 18 [50%] male) enrolled. In year 2, the MIS-C group was similar to controls on all outcome measures, except they had more somatization symptoms (Behavior Assessment Scale for Children, Third Edition mean [SD] somatization score, 52.1 [13.0] vs 46.5 [8.5]; mean difference, 5.2; 95% CI, 1.3-9.1). Within the MIS-C group, scores generally improved between initial and follow-up evaluations, a finding that was not observed in controls. Eight of 13 children with MIS-C (62%) who had abnormal neurologic examination findings in year 1 had normal examination findings by year 2. Among patients with MIS-C, measures of higher illness severity during hospitalization were associated with worse executive function in year 2 (National Institutes of Health [NIH] List Sort Working Memory Test score, −7.3 points per intensive care unit admission vs not [95% CI, −14.3 to −0.3 points] and −5.8 points per LVEF category change [95% CI, −9.1 to −2.6 points]; verbal fluency switching score, −0.8 points per LVEF category change [95% CI, −1.5 to −0.1 points]).

**Conclusions and Relevance:**

In this longitudinal, matched cohort study of children with MIS-C and controls followed up sequentially up to 2 years after hospital discharge, children with MIS-C had more somatic symptoms than control children. Overall, however, patients with MIS-C had improved neurologic and psychological outcomes between the testing intervals, performing similarly to controls on most measures by year 2 follow-up. These findings suggest that these concerns may improve over time.

## Key Points

**Question:**

Do neuropsychological sequelae observed 1 year after multisystem inflammatory syndrome in children (MIS-C) persist up to 2 years?

**Findings:**

In this cohort study of 59 participants examined serially up to 2 years after MIS-C hospitalization, cognition, behavior, and quality-of-life scores improved, and by 2 years most scores were comparable to those of 36 sibling and community controls. Lower 2-year executive functioning scores were associated with intensive care unit admission and lower left ventricular ejection fraction during MIS-C hospitalization.

**Meaning:**

Neurologic and psychological sequelae after hospitalization for MIS-C improved by 2 years after discharge, but adverse impacts of more severe acute illness may linger in some domains.

## Teaser

This cohort study examines neurologic, psychological, and quality-of-life sequelae associated with multisystem inflammatory syndrome in children.

## Introduction

Multisystem inflammatory syndrome in children (MIS-C) is a rare but potentially life-threatening complication of SARS-CoV-2 infection.^1,2,3^ Inflammation and multiorgan dysfunction are hallmarks, including acute neurologic symptoms, such as mental status change, seizures, and neuroinflammation.^4,5,6,7^ Although the incidence of MIS-C has decreased, cases are expected to continue to occur in association with increases in COVID-19 activity, particularly among unvaccinated children or those with waning immunity.^8^ Nearly 10 000 US children have been diagnosed with MIS-C,^9^ and many more experience symptoms that occur for 3 months or more after SARS-CoV-2 infection, referred to as post–COVID-19 conditions or long COVID,^10,11,12^ highlighting the importance of understanding long-term sequelae.

Few reports have assessed long-term neurologic and psychological recovery in children after COVID-19, and even fewer distinguish children with MIS-C from those who were hospitalized for acute COVID-19. A survey of families whose children were hospitalized 1 to 2 years prior with acute COVID-19 or MIS-C conducted at 9 US centers found that 22% reported cognitive concerns and 28% reported emotional concerns.^13^ Among children with MIS-C, a survey found that more than half reported symptoms referable to the brain at 6-month follow-up, including 21% with worsened school performance, memory, or attention and 52% with psychological symptoms relating to socialization, behavior, anxiety, or somatization.^14^ Using direct assessment, one study in the Netherlands observed cognitive and behavioral concerns and worse quality of life 4 months after MIS-C intensive care unit (ICU) admission.^15^ Similarly, our group previously conducted psychological and neurologic assessments of children 6 to 12 months after hospitalization for MIS-C and found reduced executive functioning, increased behavioral symptoms, and lower quality of life compared with a sibling and community control group.^16^ For the current study, we followed up our existing cohort for up to 2 years and added measures of physical activity, sleep, and family stress to better understand the nature and improvement of these sequelae.

## Methods

### Participants

This longitudinal cohort study assessed children diagnosed with MIS-C from August 1, 2020, to August 31, 2021, and matched sibling and community controls, when available. Screening and recruitment methods, including a control matching algorithm, were previously published.^16^ Patients with MIS-C were required to meet the 2020 Centers for Disease Control and Prevention (CDC) MIS-C case definition^17^; be aged 5 to 20 years at hospital discharge; speak English, Spanish, or French fluently; have internet access; and be 6 years or older at first study visit. We excluded children with substantial hearing or vision impairments or intellectual disability because these conditions might interfere with remote assessment validity. We contacted individuals who participated in year 1 (6-12 months) via mail, email, or telephone and invited them to participate in an 18- to 24-month postdischarge visit (year 2). Retention efforts included seasonal newsletters and a mug. Cases unmatched in year 1 were asked again to suggest potential controls (eg, sibling who had reached eligible age) but retained in analyses regardless. Control SARS-CoV-2 testing was not performed because the focus was to identify controls within a similar environment. Based on household transmission^18,19^ and national seroprevalence studies,^20,21^ controls were likely exposed at initial identification or in the follow-up interval. The study was approved by the Central Boston Children’s Hospital institutional review board (IRB) and reviewed by participating site IRBs with CDC IRB reliance.^22^ Parents and participants 18 years or older provided verbal informed consent, and children assented. Participants received an evaluation report and an iPad (Apple [$199 value]) or a fitness tracker ($70 value) as remuneration. The study followed the Strengthening the Reporting of Observational Studies in Epidemiology (STROBE) guidelines.^23^

### Study Procedures

Year 1 assessments were previously described.^16^ The year 2 evaluation included a structured interview, parent and child surveys, remote neuropsychological assessment and neurologic examination, and sleep and activity data tracked for up to 10 days using a wearable device (FitBit Inspire 2, Google LLC; ≥5 calendar days of usable data were required for analysis) (eMethods in Supplement 1). We provided a study-issued tablet to each participant for surveys and assessments.^16^ The pediatric neuropsychologists (J.C., A.M.T., and M.M.) and board-certified pediatric neurologists (C.K.R. and a nonauthor) were blinded to group status.

#### Structured Interview

The study team conducted a structured family interview to obtain demographic and medical information, including self-reported race and ethnicity information, which was collected to evaluate potential sociodemographic-related disparities in outcomes. Race categories included Black or African American, White, Multiracial, and other (Asian, Brazilian, Middle Eastern, or declined to answer) or unknown. We calculated the Social Vulnerability Index, a measure of neighborhood social risk designed to identify at-risk communities during a public health emergency (range of 0-1, with higher values indicate greater social risk), at enrollment.^24^ The interview included baseline medical conditions, symptoms or new diagnoses since MIS-C discharge, and new symptoms or diagnoses since year 1. We inquired about rehabilitative, cognitive, or behavioral therapies received (not hospitalizations). To gather subjective general impressions, parents and adult participants rated the percent to which the patient’s current status compared with baseline health status before MIS-C hospitalization (cases) or COVID-19 exposure (controls) across 5 domains: energy, appetite, sleep, cognition, and mood (categorized as approximately 0% [indicating no improvement], 25%, 50%, 75%, 90%, 100%, or unknown). From hospital records, we extracted MIS-C hospitalization–related medical data, including length of stay (hospital and ICU), lowest left ventricular ejection fraction (LVEF) category (normal or mild [45%-54%], moderate [35%-44%], or severe [<35%] depression), neurologic involvement at presentation, inpatient corticosteroid use, cardiopulmonary resuscitation, shock requiring vasopressor support, extracorporeal membranous oxygenation, neurologic examination at discharge, and readmission within 30 days as described previously.^16^

#### Surveys

Parents completed the Behavior Rating Inventory of Executive Function, Second Edition (BRIEF-2)^25^ or adult version of BRIEF^26^ (for those older than 18 years) to assess executive functioning in daily life and the Behavior Assessment Scale for Children, Third Edition (BASC-3)^27^ to assess behavioral functioning. We obtained parent- and self-report Pediatric Quality of Life Inventory (PedsQL) 4.0 Generic Core Scale scores^28^ and Patient-Reported Outcomes Measurement Information System (PROMIS) sleep-related impairment, sleep disturbance (8 items each),^29^ and global health scale scores. For parental mental health, parents completed the self-reported Depression, Anxiety, and Stress Scale 21 (DASS-21),^30^ with each question rated on a 4-point scale (with 0 indicating that the statement did not apply at all and 3 indicating the statement applied very much or most of the time) based on the past week. Children completed the self-reported Beck Anxiety Inventory and the Beck Self-Concept Inventory from the Beck Youth Inventory, Second Edition^31^ (substituting Beck Anxiety Inventory^32^ for those older than 18 years).

#### Neuropsychological Assessment

Neuropsychological assessment included measures of executive functioning, with the NIH Cognition Toolbox List Sort Working Memory Test score as the primary outcome, supplemented by the Working Memory Index from the Wechsler Intelligence Scale for Children, Fifth Edition^33^ or the Wechsler Adult Intelligence Scale IV scores,^34^ and the verbal fluency switching and color-word interference switching scores from the Delis-Kaplan Executive Function System^35^ to assess inhibitory control and cognitive flexibility. We used the age-appropriate Wechsler Scale Processing Speed Index for processing speed (130 or above: very superior; 120-129: superior; 110-119: high average; 90-109: average; 80-89: low average; 70-79: borderline; 70 or below: extremely low). As global cognitive ability generally remains stable over time,^36^ an estimate of general cognitive ability was obtained only for participants without year 1 data, using the 2-subtest version of the Wechsler Abbreviated Scale of Intelligence, Second Edition.^37^

#### Neurologic Examination

New participants and those with abnormal year 1 examination findings underwent a standardized remote neurologic examination, including cranial nerves, motor, coordination, and gait maneuvers. Overall clinical impression was dichotomized as normal vs abnormal for analysis.^16^

### Statistical Analysis

Among patients with MIS-C with year 1 evaluations, we compared those who returned with those who did not (ie, lost to follow-up) on demographic variables, baseline medical conditions, and year 1 outcome measures using 2-tailed *t*, Fisher exact, or χ^2^ tests. We compared year 2 survey results and psychological scores between the MIS-C and control groups using linear or logistic regression with generalized estimating equations using the exchangeable working assumption, which accounts for clustering within matched pairs as well as inclusion of MIS-C cases without a matched control. Among those with longitudinal data, we performed change score analysis of repeated measures within each group, using similar regression techniques and assumptions. We explored whether demographic and medical variables associated with year 1 outcomes retained significance in year 2, using univariable linear and logistic regression. We also examined whether newly acquired data on parent anxiety, stress, or depression (categorized as moderate or higher in ≥1 domain vs not) or total recorded sleep duration and activity (mean steps per day) on the wearable device were associated with outcomes. Statistical significance was assessed with 95% CIs without multiple comparisons correction given that analyses were exploratory and conducted using R Studio, version 3 (R Project for Statistical Computing). Data analysis was performed from May 2024 to January 2025.

## Results

### Demographic and Medical Data

Overall, 95 participants were included in the study; 93 of 108 participants (86%) returned from the year 1 study and 2 participants were added in year 2 (median [IQR] age, 12.6 [11.0-15.7] years; 38 [40%] female and 57 [60%] male; 15 Black or African American, 58 White, 10 multiracial, and 12 other or unknown). From the original cohort, 59 of 64 patients with MIS-C (92%) (mean [SD] age, 13.2 [4.0] years; 39 [66%] male and 20 [34%] female) reenrolled for year 2 alongside 36 controls (mean [SD] age, 13.5 [3.5] years; 18 [50%] male and 18 [50%] female), comprising 34 of 44 original participants (77%) and 2 new controls for previously unmatched MIS-C cases (Table 1). Controls included 32 siblings, 3 half-siblings, and 1 cousin. Median (IQR) duration between discharge and evaluation was 23.9 (22.1-25.4) months.

**Table 1.** Demographic and Medical Characteristics of Children 2 Years After MIS-C Hospitalization and Age-Matched Controls

Characteristic^b^	Participants, No. (%) (N = 95)^a^	
	Patients with MIS-C (n = 59)	Control group (n = 36)
Age, median (IQR), y	12.1 (10.2-15.5)	13.0 (11.8-15.9)
Sex		
Female	20 (34)	18 (50)
Male	39 (66)	18 (50)
Race		
Black or African American	12 (20)	3 (8)
White	35 (59)	23 (64)
Multiracial	5 (8)	5 (14)
Other or unknown^c^	7 (12)	5 (14)
Hispanic ethnicity	9 (15)	4 (11)
Social Vulnerability Index, median (IQR)	0.36 (0.20-0.60)	0.33 (0.13-0.59)
Primary caregiver education		
Graduated college or greater	36 (61)	24 (67)
Some college	13 (22)	7 (19)
Grade 12 or less	10 (17)	5 (14)
Insurance		
Private insurance	40 (70)	29 (81)
Self-pay	3 (5)	1 (3)
Government insurance	14 (25)	6 (17)
Single family home	50 (85)	33 (92)
Baseline medical conditions	16 (27)	6 (17)
Neurologic^d^	1 (2)	1 (3)
Respiratory^e^	6 (10)	5 (14)
Gastrointestinal or hepatic	4 (7)	0
Endocrine	4 (7)	0
Obesity	7 (12)	3 (8)
Other^f^	3 (5)	1 (3)
Baseline psychological conditions^g^	10 (17)	4 (11)

Abbreviation: MIS-C, multisystem inflammatory syndrome in children.

^a^ Unless otherwise indicated.

^b^ Data complete except for Social Vulnerability Index (56 patients with MIS-C and 33 controls), private insurance (57 patients with MIS-C and 36 controls), and baseline medical and psychological conditions (58 patients with MIS-C and 35 controls).

^c^ Other or unknown race includes self-reported as Asian (3 patients with MIS-C and 2 controls), Brazilian (1 patient with MIS-C and 1 control), Middle Eastern (1 patient with MIS-C case and 1 control), or declined to answer (2 patients with MIS-C and 1 control).

^d^ Dopa-responsive dystonia (1 patient with MIS-C), and Guillan-Barre syndrome (1 control).

^e^ Asthma (6 patients with MIS-C and 4 controls) and obstructive sleep apnea (1 control).

^f^ Osteochondritis dissecans (1 patient with MIS-C), horseshoe kidney (1 patient with MIS-C), aortic valve abnormality (1 patient with MIS-C), and intermittent exotropia (1 control).

^g^ Attention-deficit/hyperactivity disorder (4 patients with MIS-C and 2 controls), anxiety (1 patient with MIS-C and 1 control), autism (1 patient with MIS-C and 1 control), attention-deficit/hyperactivity disorder and dyslexia (1 patient with MIS-C), attention-deficit/hyperactivity disorder and autism (1 patient with MIS-C), attention-deficit/hyperactivity disorder, autism, and anxiety (1 patient with MIS-C), and anxiety and posttraumatic stress disorder (1 patient with MIS-C).

Participants who returned in year 2 were similar to those who did not with respect to most demographic variables, baseline medical conditions, MIS-C hospitalization characteristics, and year 1 cognitive and behavioral measure scores. Nonreturners had lower socioeconomic status than returners on some measures and a longer length of hospital stay and higher rate of shock or need for cardiopulmonary resuscitation (eTable 1 in Supplement 1). eTable 2 in Supplement 1 reports therapeutic services received between years 1 and 2.

### MIS-C vs Control Group Comparison

Of 59 cases and 36 controls who responded to the general impressions of health status survey, fewer cases than controls had returned to their pre–COVID-19 baseline for energy (worse than baseline; energy: 15 of 58 [26%] vs 4 of 36 [11%]; appetite: 9 of 58 [16%] vs 0 of 36 [0%]; sleep: 19 of 58 [33%] vs 2 of 35 [6%]; cognition: 17 of 58 [29%] vs 2 of 35 [6%]; mood: 16 of 58 [28%] vs 5 of 36 [14%]) (eTable 3 in Supplement 1). The MIS-C and control groups had comparable scores on nearly all assessments, including the NIH List Sort Working Memory Test, BASC-3, and PedsQL self-reported psychosocial health (Table 2), for which each had shown group differences in year 1.^16^ The MIS-C group had more somatization symptoms (eg, headache and stomach pain) than the control group (BASC-3 somatization mean [SD] score, 52.1 [13.0] vs 46.5 [8.5]; mean difference, 5.2; 95% CI, 1.3-9.1). The Beck Youth Inventory anxiety and self-concept scores were similar between groups, as were the total sleep and activity scores. Adjustment for sex did not appreciably alter findings.

**Table 2.** Psychological Outcomes at 2-Year Follow-Up by Group and Comparison of Children 2 Years After MIS-C Hospitalization and Age-Matched Controls

Outcome^b^	Score, mean (SD)		Patients with MIS-C vs controls (95% CI)^a^	
	Patients with MIS-C (n = 59)	Controls (n = 36)	Mean difference	Standardized mean difference^c^
Wechsler Intelligence Scales (WASI-II, WISC-V, and WAIS-IV)				
IQ estimate	101.3 (14.6)	103.2 (14.0)	−2.2 (−7.0 to 2.5)	−0.15 (−0.47 to 0.17)
Working Memory Index	102.4 (14.1)	104.1 (14.4)	−1.5 (−6.3 to 3.2)	−0.10 (−0.42 to 0.21)
Processing Speed Index	100.9 (13.3)	97.2 (13.1)	3.3 (−2.7 to 9.3)	0.22 (−0.18 to 0.62)
National Institutes of Health Cognition Toolbox				
List Sort Working Memory Test	102.1 (13.8)	103.1 (14.7)	−0.8 (−5.3 to 3.7)	−0.05 (−0.35 to 0.25)
Delis-Kaplan Executive Function System				
Verbal fluency switching	10.5 (2.9)	11.6 (3.2)	−1.1 (−2.3 to 0.2)	−0.36 (−0.78 to 0.06)
Color-word interference switching	10.1 (2.8)	10.5 (2.5)	−0.5 (−1.6 to 0.7)	−0.15 (−0.55 to 0.24)
BRIEF global executive composite^d^	50.5 (11.7)	50.1 (11.6)	0.3 (−3.2 to 3.7)	0.03 (−0.32 to 0.37)
Behavior Assessment Scale for Children, Third Edition				
Internalizing problems	49.7 (11.9)	46.0 (9.6)	3.0 (−0.4 to 6.4)	0.30 (−0.04 to 0.64)
Anxiety	47.9 (11.0)	47.8 (10.9)	−0.4 (−4.4 to 3.6)	−0.04 (−0.44 to 0.36)
Depression	49.1 (9.9)	45.8 (9.5)	2.9 (−0.1 to 6.0)	0.29 (−0.01 to 0.60)
Somatization	52.1 (13.0)	46.5 (8.5)	5.2 (1.3 to 9.1)	0.52 (0.13 to 0.91)
Externalizing problems	48.5 (9.6)	46.2 (8.9)	2.5 (−0.7 to 5.6)	0.25 (−0.07 to 0.56)
Hyperactivity	49.6 (11.8)	47.0 (10.7)	2.8 (−1.1 to 6.8)	0.28 (−0.11 to 0.68)
Aggression	49.2 (8.9)	47.4 (8.2)	1.8 (−1.3 to 4.8)	0.18 (−0.13 to 0.48)
Conduct problems	47.2 (7.0)	44.9 (7.6)	2.4 (−0.4 to 5.2)	0.24 (−0.04 to 0.52)
Behavioral symptoms index^e^	47.8 (11.1)	45.6 (10.9)	2.2 (−1.1 to 5.5)	0.22 (−0.11 to 0.55)
Atypicality	48.0 (11.8)	44.9 (6.5)	3.0 (−0.1 to 6.1)	0.30 (−0.01 to 0.61)
Withdrawal	48.6 (11.3)	47.9 (12.7)	0.3 (−4.1 to 4.7)	0.03 (−0.41 to 0.47)
Attention problems	48.5 (13.5)	46.1 (12.4)	2.2 (−2.3 to 6.7)	0.22 (−0.23 to 0.67)
Adaptive skills composite	52.5 (10.4)	53.4 (11.5)	−1.0 (−4.7 to 2.7)	−0.10 (−0.47 to 0.27)
PedsQL Generic Core Scale				
Self-report total	81.0 (15.4)	84.5 (13.4)	−2.7 (−7.5 to 2.2)	−0.20 (−0.57 to 0.17)
Physical health	84.6 (17.0)	89.6 (12.9)	−4.6 (−10.0 to 0.9)	−0.33 (−0.72 to 0.07)
Psychosocial health	80.8 (18.4)	85.0 (18.4)	−2.4 (−8.6 to 3.8)	−0.16 (−0.58 to 0.26)
Parent-report total	83.0 (16.9)	88.4 (13.4)	−4.5 (−9.3 to 0.3)	−0.28 (−0.58 to 0.02)
Physical health	86.2 (20.4)	90.3 (16.9)	−2.9 (−8.4 to 2.5)	−0.21 (−0.60 to 0.18)
Psychosocial health	81.2 (17.6)	87.5 (14.5)	−5.4 (−11.0 to 0.1)	−0.37 (−0.75 to 0.01)
PROMIS sleep disturbance	50.8 (9.6)	49.3 (9.2)	1.4 (−2.6 to 5.3)	0.14 (−0.26 to 0.53)
PROMIS sleep impairment	49.5 (9.7)	48.3 (8.7)	1.2 (−1.9 to 4.4)	0.12 (−0.19 to 0.44)
PROMIS global health self-report	51.3 (9.2)	53.9 (10.0)	−2.0 (−5.7 to 1.7)	−0.20 (−0.57 to 0.17)
PROMIS global health parent-report	53.6 (10.1)	56.3 (9.6)	−1.8 (−5.0 to 1.4)	−0.18 (−0.50 to 0.14)
Beck Youth Inventory self-concept	53.0 (9.6)	55.8 (9.2)	−2.1 (−5.6 to 1.5)	−0.21 (−0.56 to 0.15)
Beck Anxiety Inventory	47.2 (10.7)	46.1 (8.1)	0.8 (−2.6 to 4.2)	0.08 (−0.26 to 0.42)
Fitness tracker steps, 1000/d	9.6 (4.5)	9.5 (4.1)	0.2 (−1.4 to 1.7)	NA
Fitness tracker total sleep, h/d	7.4 (0.8)	7.4 (0.8)	0.0 (−0.2 to 0.3)	NA

Abbreviations: BRIEF, Behavior Rating Inventory of Executive Function; MIS-C, multisystem inflammatory syndrome in children; NA, not applicable; PedsQL, Pediatric Quality of Life Inventory; PROMIS, Patient-Reported Outcomes Measurement Information System; WAIS-IV, Wechsler Adult Intelligence Scale, Fourth Edition; WASI-II, Wechsler Abbreviated Scale of Intelligence, Second Edition; WISC-V, Wechsler Intelligence Scale for Children, Fifth Edition.

^a^ Adjusted for matched pairs.

^b^ Number of participants completing each assessment are as follows: IQ estimate, 59 patients with MIS-C and 36 controls; Working Memory Index, 58 patients with MIS-C and 36 controls; Processing Speed Index, 53 patients with MIS-C and 33 controls (resulting from nonreturn of mailed booklets); List Sort Working Memory Test, 57 patients with MIS-C and 36 controls; verbal fluency switching, 53 of 55 patients with MIS-C and 34 of 34 controls (given to participants aged ≥8 years); color-word interference switching, 52 of 55 patients with MIS-C and 34 of 34 controls (given to participants aged ≥8 years); BRIEF, 57 patients with MIS-C and 35 controls; Behavior Assessment Scale for Children, Third Edition, 52 patients with MIS-C and 35 controls (except behavioral symptoms index and atypicality have 51 patients with MIS-C and 35 controls); PedsQL self-report, 58 patients with MIS-C and 35 controls; PedsQL parent-report, 52 patients with MIS-C and 35 controls; PROMIS, 54 patients with MIS-C and 33 controls; PROMIS global health self-report, 46 patients with MIS-C and 29 controls; PROMIS global health parent-report, 49 patients with MIS-C and 31 controls; Beck Youth Inventory self-concept, 51 of 51 patients with MIS-C and 30 of 32 controls (given to participants aged ≤18 years); Beck Anxiety Inventory, 57 patients with MIS-C and 33 controls; fitness tracker total steps, 50 patients with MIS-C cases and 32 controls; fitness tracker total sleep, 37 patients with MIS-C cases and 25 controls.

^c^ Standardized mean difference is the effect size relative to 1 SD based on test parameters or normative data.

^d^ Includes second edition or adult version of BRIEF.

^e^ Includes depression, hyperactivity, aggression, atypicality, withdrawal, and attention.

### Change Score Analysis

Comparing year 2 with year 1 (Table 3; eFigures 1-3 in Supplement 1), executive functioning scores improved in patients with MIS-C (NIH List Sort Working Memory Test mean difference, 6.0; 95% CI, 2.2-9.8]; Delis-Kaplan Executive Function System color-word interference switching subtest mean difference, 1.1; 95% CI, 0.3-1.9). The MIS-C group also had fewer internalizing symptoms (BASC-3 internalizing problems difference, −3.6; 95% CI, −6.0 to −1.1) (Figure), reflecting less anxiety, depression, and somatization in year 2. Parent-reported psychosocial quality of life also improved (PedsQL Generic Core Scale mean difference, 4.7; 95% CI, 0.5-8.9) and sleep disturbance decreased (PROMIS sleep disturbance mean difference, −3.1; 95% CI, −6.1 to −0.2). For controls, year 1 and 2 scores were comparable in cognitive, quality of life, and sleep domains; however, the control group had lower (better) atypicality (ie, behaviors perceived as odd; BASC-3 atypicality mean difference, −3.5; 95% CI, −6.2 to −0.7) scores in year 2. Among the 13 of 59 children with MIS-C who had an abnormal neurologic examination general impression in year 1 and were thus reexamined in year 2, 5 still had abnormal general impressions (predominantly motor findings).

**Table 3.** Changes in Psychological Outcomes by Group for Children 2 Years After MIS-C Hospitalization and Age-Matched Controls

Outcome^b^	Year 2 vs year 1 (95% CI)^a^				
	Patients with MIS-C		Controls		
	Mean difference	Standardized mean difference^c^	Mean difference	Standardized mean difference^c^
Wechsler Intelligence Scales (WASI-II, WISC-V, and WAIS-IV)				
Working Memory Index	2.8 (−0.2 to 5.8)	0.19 (−0.02 to 0.39)	1.8 (−2.1 to 5.6)	0.12 (−0.14 to 0.38)
Processing Speed Index	3.1 (−0.7 to 6.9)	0.21 (−0.05 to 0.46)	0.6 (−3.1 to 4.3)	0.04 (−0.21 to 0.29)
National Institute of Health Cognition Toolbox				
List Sort Working Memory Test	6.0 (2.2 to 9.8)	0.40 (0.15 to 0.65)	−0.2 (−4.6 to 4.3)	−0.01 (−0.31 to 0.29)
Delis-Kaplan Executive Function System				
Verbal fluency switching	−0.2 (−1.0 to 0.7)	−0.06 (−0.34 to 0.23)	0.8 (−0.4 to 2.1)	0.28 (−0.15 to 0.70)
Color-word interference switching	1.1 (0.3 to 1.9)	0.37 (0.11 to 0.63)	0.8 (0.0 to 1.6)	0.26 (−0.01 to 0.52)
BRIEF global executive composite^d^	−1.6 (−3.9 to 0.7)	−0.16 (−0.39 to 0.07)	0.3 (−2.6 to 3.2)	0.03 (−0.26 to 0.32)
Behavior Assessment Scale for Children, Third Edition				
Internalizing problems	−3.6 (−6.0 to −1.1)	−0.36 (−0.60 to −0.11)	−1.3 (−3.3 to 0.7)	−0.13 (−0.33 to 0.07)
Anxiety	−2.4 (−4.7 to −0.2)	−0.24 (−0.47 to −0.02)	−1.6 (−4.6 to 1.4)	−0.16 (−0.46 to 0.14)
Depression	−3.2 (−5.5 to −1.0)	−0.32 (−0.55 to −0.10)	−2.1 (−4.4 to 0.1)	−0.21 (−0.44 to 0.01)
Somatization	−3.4 (−6.5 to −0.2)	−0.34 (−0.65 to −0.02)	0.1 (−2.5 to 2.7)	0.01 (−0.25 to 0.27)
Externalizing problems	−1.5 (−3.6 to 0.7)	−0.15 (−0.36 to 0.07)	−0.7 (−2.2 to 0.8)	−0.07 (−0.22 to 0.08)
Hyperactivity	−0.8 (−3.4 to 1.9)	−0.08 (−0.34 to 0.19)	−0.2 (−2.5 to 2.0)	−0.02 (−0.25 to 0.20)
Aggression	−1.4 (−3.6 to 0.8)	−0.14 (−0.36 to 0.08)	−0.7 (−2.5 to 1.1)	−0.07 (−0.25 to 0.11)
Conduct problems	−1.7 (−3.6 to 0.1)	−0.17 (−0.36 to 0.01)	−1.7 (−3.5 to 0.1)	−0.17 (−0.35 to 0.01)
Behavioral symptoms index^e^	−2.1 (−4.4 to 0.1)	−0.21 (−0.44 to 0.01)	−1.5 (−3.7 to 0.6)	−0.15 (−0.37 to 0.06)
Atypicality	−0.7 (−2.7 to 1.2)	−0.07 (−0.27 to 0.12)	−3.5 (−6.2 to −0.7)	−0.35 (−0.62 to −0.07)
Withdrawal	−1.5 (−3.5 to 0.5)	−0.15 (−0.35 to 0.05)	−0.4 (−3.6 to 2.7)	−0.04 (−0.36 to 0.27)
Attention problems	−0.9 (−3.7 to 1.8)	−0.09 (−0.37 to 0.18)	−0.6 (−3.8 to 2.6)	−0.06 (−0.38 to 0.26)
Adaptive skills composite	2.2 (−0.4 to 4.9)	0.22 (−0.04 to 0.49)	0.8 (−2.0 to 3.6)	0.08 (−0.20 to 0.36)
PedsQL generic				
Self-report total	0.8 (−2.4 to 4.0)	0.06 (−0.18 to 0.31)	−1.5 (−5.7 to 2.7)	−0.11 (−0.43 to 0.20)
Physical health	−1.3 (−5.5 to 2.9)	−0.09 (−0.39 to 0.21)	−0.5 (−5.2 to 4.1)	−0.04 (−0.37 to 0.30)
Psychosocial health	3.8 (−0.3 to 8.0)	0.26 (−0.02 to 0.54)	1.4 (−4.2 to 7.0)	0.10 (−0.28 to 0.48)
Parent-report total	3.0 (−0.7 to 6.7)	0.19 (−0.05 to 0.42)	−0.5 (−4.2 to 3.2)	−0.03 (−0.27 to 0.20)
Physical health	−0.3 (−4.7 to 4.1)	−0.02 (−0.34 to 0.29)	−1.8 (−4.7 to 1.1)	−0.09 (−0.23 to 0.05)
Psychosocial health	4.7 (0.5 to 8.9)	0.3 (0.03 to 0.56)	0.0 (−4.8 to 4.8)	0.00 (−0.30 to 0.30)
PROMIS sleep disturbance	−3.1 (−6.1 to −0.2)	−0.31 (−0.61 to −0.02)	−1.3 (−4.3 to 1.6)	−0.13 (−0.43 to 0.16)
PROMIS sleep impairment	0.0 (−2.8 to 2.7)	0.0 (−0.28 to 0.27)	0.2 (−2.9 to 3.3)	0.02 (−0.29 to 0.33)

Abbreviations: BRIEF, Behavior Rating Inventory of Executive Function; MIS-C, multisystem inflammatory syndrome in children; PedsQL, Pediatric Quality of Life Inventory; PROMIS, Patient-Reported Outcomes Measurement Information System; WAIS-IV, Wechsler Adult Intelligence Scale, Fourth Edition; WASI-II, Wechsler Abbreviated Scale of Intelligence, Second Edition; WISC-V, Wechsler Intelligence Scale for Children, Fifth Edition.

^a^ Adjusted for matched pairs.

^b^ Number of participants completing each assessment in year 2 are listed in Table 2. The number of participants completing each assessment in year 1 was previously published.^10^

^c^ Standardized mean difference is the effect size relative to 1 SD based on test parameters or normative data in the linear regression analyses using generalized estimating equations.

^d^ Includes second edition or adult version of BRIEF.

^e^ Includes depression, hyperactivity, aggression, atypicality, withdrawal, and attention.

**Figure.**
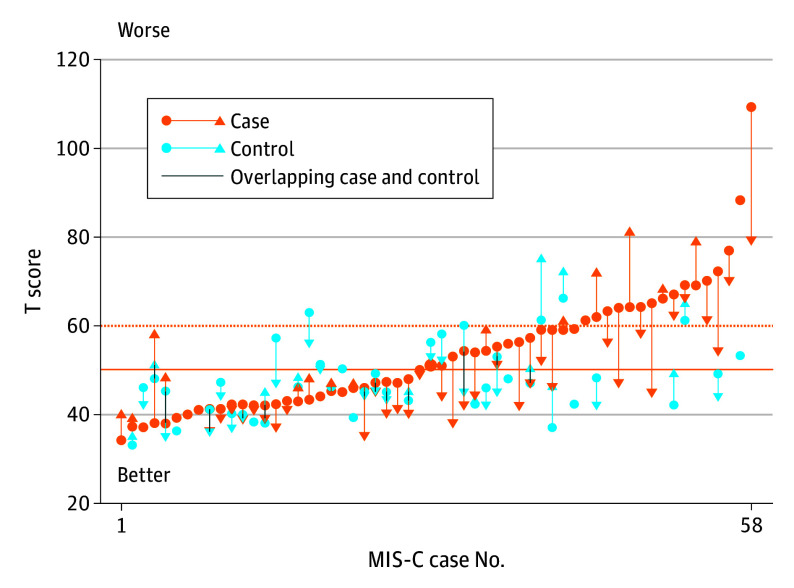
Group Differences and 2-Year Change in Behavior Assessment Scale for Children, Third Edition Internalizing Problems Scores The figure shows the year 1 and year 2 scores for 58 patients with multisystem inflammatory syndrome in children (MIS-C) and 39 controls sorted from lowest to highest scores. Lines and arrow reflect the change between year 1 and year 2 scores, where available. Matched controls, where available, are plotted on the same x-axis value as the cases. Solid orange horizontal line reflects the test mean. Dotted orange horizontal line indicates 1 SD worse than the mean; the test defined at-risk threshold.

### Demographic, Medical, and Psychological Associations With Outcome in the MIS-C Group

Social Vulnerability Index, lowest LVEF category, ICU stay (dichotomized as yes or no), and neurologic symptoms at hospital presentation showed significant associations with outcomes in year 1 and were thus reexplored in year 2. In year 2, lower scores in aspects of executive functioning continued to be associated with left ventricular dysfunction and ICU admission, although the specific outcome measure associations differed from year 1^16^ (NIH List Sort Working Memory Test, −5.8 points per change in LVEF category [95% CI, −9.1 to −2.6 points] and −7.3 points per ICU admission vs not [95% CI, −14.3 to −0.3 points]; verbal fluency switching, −0.8 points per change in LVEF category [95% CI, −1.5 to −0.1 points]). Similarly, neurologic symptoms at hospital presentation continued to be associated with fewer adverse behavioral symptoms (BASC-3 behavioral symptoms index mean difference, −8.0; 95% CI, −13.9 to −2.1).

Data newly obtained in year 2, including participant total sleep and daily activity (steps) and parent and child mental health, revealed that higher daily activity was associated with fewer parent-observed somatic symptoms (BASC-3 somatization mean difference, −0.8; 95% CI, −1.6 to 0.0), whereas higher parent self-reported stress, anxiety, or depression on the DASS-21 was associated with worse scores on the child’s BASC-3 behavioral symptoms index (mean difference, 10.2; 95% CI, 6.2-14.2).

## Discussion

In this longitudinal matched cohort study, we examined neurologic, psychological, and quality-of-life outcomes in patients with MIS-C 2 years after hospitalization and compared them with a sibling and community control group. We explored associations of demographic and hospitalization-related medical data with outcomes and examined newly acquired sleep and activity data and parent mental health to assess potential mechanisms. Generally, outcomes in patients with MIS-C improved from 1 to 2 years after MIS-C, although parents continued to report observing more somatic symptoms in patients with MIS-C than in controls. Greater illness severity at hospitalization, measured by cardiac dysfunction and ICU admission, was associated with worse executive functioning in year 2. More daily activity and less parental stress were associated with fewer behavioral symptoms after MIS-C. Overall, the results suggest an improving trajectory in the children with MIS-C on the measures assessed and provide insight into potential mechanisms underlying these concerns.

Our findings, based on neuropsychological and neurologic evaluation of patients, extend knowledge about recovery after MIS-C. Data from the present cohort^16^ and others^15,38,39,40,41^ suggest more cognitive and behavioral symptoms and lower quality of life in children with MIS-C compared with controls or healthy individuals up to 1 year after hospitalization. We evaluated participants for 2 years. Children with MIS-C continued to experience more parent-reported somatic symptoms than controls, but scores improved across most domains, including executive functioning, internalizing symptoms (which included anxiety, depression, and somatization), sleep, and quality of life. These findings align with those from a clinic-based report demonstrating few symptoms at 2 years.^42^ Improvements within the MIS-C group from 1- to 2-year follow-up were not mirrored in the control group. These disparate trajectories are consistent with the hypothesis that MIS-C caused transient neurologic and psychological effects in the MIS-C group separate from broader effects of COVID-19–related societal restrictions.

The data also provide insight into potential mechanisms impacting neurologic and psychological recovery after MIS-C. Although there was variability in the specific outcome measures showing associations, in both year 1^16^ and year 2, cardiac dysfunction and ICU admission were associated with worse executive functioning scores within the MIS-C group. These associations may relate to evolution of post–intensive care syndrome in pediatrics, a conceptual framework describing cognitive, physical, and mental health impairments after serious illness necessitating ICU admission.^43^ In our cohort, neurologic findings at presentation or follow-up examination, which may reflect structural brain changes, were not associated with cognitive outcomes, recognizing limitations of small sample size and remote assessment. Future neuroimaging studies are needed to assess whether there are structural brain changes in patients with MIS-C that correlate with hospitalization-related variables and cognitive outcomes.

We hypothesized that environmental factors might contribute to neurologic and psychological recovery after MIS-C. For example, early activity restrictions may lead to longer-term changes in activity or sleep and thereby impact functioning and recovery. Activity and sleep findings, newly collected in year 2, were similar between the 2 groups and were generally not associated with 2-year outcomes, although more activity was associated with fewer somatic symptoms. Similarly, elevated parental stress, anxiety, or depression showed limited association with outcomes. It is possible that measuring these factors earlier in recovery would have been more sensitive to detect an association. Alternatively, these factors may not be major contributors to long-term outcomes.

MIS-C is one of the postacute sequelae that may occur in some children after SARS-CoV-2 infection,^12^ and the cognitive concerns that persist after MIS-C observed in our cohort have some overlap with those reported among individuals with neurologic manifestations after acute COVID-19 (eg, cognitive difficulties, internalizing symptoms, and sleep disturbance).^44^ The improvement over 2 years in neurologic and psychological symptoms in our cohort with MIS-C is also similar to reported improvement in cognitive symptoms in youth affected by long COVID.^45^ Although findings in our cohort may have relevance for the broader spectrum of post–COVID-19 conditions, the mechanisms underlying neuropsychological impairments are not yet well understood, and different pathways may lead to similar symptoms in these 2 conditions. Nonetheless, our findings of improvement in neurologic, psychological, and quality-of-life scores 2 years after hospitalization provide reason for optimism in the MIS-C population.

### Limitations

This study has limitations. First, children with preexisting or new neurologic or psychological symptoms may have been more likely to participate or return for follow-up. Despite this potential source of selection bias, which would favor worse outcomes, the scores of patients with MIS-C improved from 1- to 2-year follow-up. Second, although we used strategies to optimize ease of participation (eg, flexible hours and remote administration), the cohort is predominantly White, English-speaking, and socioeconomically advantaged relative to the overall North American population. In addition, children of higher socioeconomic status and lower illness severity were more likely to return for year 2 than those of lower socioeconomic status. Overall, 92% of MIS-C participants and 77% of controls returned, minimizing the impact of loss to follow-up. Third, the examination was conducted remotely, limiting neurologic examination sensitivity, and we were not able to assess a full range of neurocognitive skills due to technical limitations of administration (eg, response time). Studies that include in-person evaluation may provide additional insight into these areas. We were not able to quantify the impact of any treatment received given the heterogeneity of conditions and therapies. Fourth, although the study was noninterventional, children may have experienced a benefit from year 1 participation that contributed to the positive trajectory, although this finding would be expected among both groups.

## Conclusions

In this cohort study of children with MIS-C 2 years after hospital discharge, children hospitalized for MIS-C performed similarly to matched control participants on most measures. Participants with MIS-C also showed improvements in aspects of executive functioning, internalizing problems (anxiety, depression, and somatization), quality of life, and sleep problems that were not seen in the matched control sample. Parents continued to report more somatization symptoms in the patients with MIS-C than controls (despite improvement in somatization between year 1 and year 2). The findings suggest that neuropsychological status improves over time for patients with MIS-C. However, worse executive functioning (particularly working memory) continued to be associated with worse cardiac function during hospitalization and ICU stay. Neuroimaging to evaluate structural brain measures that may correlate with these cognitive dimensions in MIS-C is warranted.

## Notes

Supplement 1. **eMethods.** Supplemental Methods **eTable 1.** Characteristics of MIS-C Cases Returning vs Non-Returning at Year 2 **eTable 2.** Services Received by MIS-C Cases and Controls Between Year 1 and Year 2 **eTable 3.** General Impressions of Health Status at Year 2 Relative to Baseline in Children 2 Years After MIS-C Hospitalization and Age-Matched Controls **eFigure 1.** Parallel Line Plot Depicting Group Differences and 2-Year Change in National Institute of Health Cognition Toolbox List Sort Working Memory **eFigure 2.** Parallel Line Plot Depicting Group Differences and 2-Year Change in PedsQL generic self-report total score **eFigure 3.** General Impressions of Health Status at Year 2 Relative to Baseline in Children 2 Years After MIS-C Hospitalization and Age-Matched Controls

Supplement 2. **Nonauthor Collaborators.** The Overcoming COVID-19 Investigators

Supplement 3.
Data Sharing Statement
