# Supplementary material for: Neurologic and Psychological Outcomes 2 Years After Multisystem Inflammatory Syndrome in Children
Source: JAMA Netw Open. 2025 Jun 2;8(6):e2512487. doi: 10.1001/jamanetworkopen.2025.12487 (PMC12131094; doi:10.1001/jamanetworkopen.2025.12487)
Supplement: Supplement 1. — eMethods. Supplemental Methods eTable 1. Characteristics of MIS-C Cases Returning vs Non-Returning at Year 2 eTable 2. Services Received by MIS-C Cases and Controls Between Year 1 and Year 2 eTable 3. General Impressions of Health Status at Year 2 Relative to Baseline in Children 2 Years After MIS-C Hospitalization and Age-Matched Controls eFigure 1. Parallel Line Plot Depicting Group Differences and 2-Year Change in National Institute of Health Cognition Toolbox List Sort Working Memory eFigure 2. Parallel Line Plot Depicting Group Differences and 2-Year Change in PedsQL generic self-report total score eFigure 3. General Impressions of Health Status at Year 2 Relative to Baseline in Children 2 Years After MIS-C Hospitalization and Age-Matched Controls [file jamanetwopen-e2512487-s001.pdf]

Supplemental Online Content

Rollins CK, Wypij D, Zambrano LD, et al. Neurologic and psychological outcomes 2 years after multisystem inflammatory syndrome in children. *JAMA Netw Open*. 2025;8(5):e2512487. doi:10.1001/jamanetworkopen.2025.12487

eMethods. Supplemental Methods
eTable 1. Characteristics of MIS-C Cases Returning vs Non-Returning at Year 2
eTable 2. Services Received by MIS-C Cases and Controls Between Year 1 and Year 2
eTable 3. General Impressions of Health Status at Year 2 Relative to Baseline in Children 2 Years After MIS-C Hospitalization and Age-Matched Controls
eFigure 1. Parallel Line Plot Depicting Group Differences and 2-Year Change in National Institute of Health Cognition Toolbox List Sort Working Memory
eFigure 2. Parallel Line Plot Depicting Group Differences and 2-Year Change in PedsQL generic self-report total score
eFigure 3. General Impressions of Health Status at Year 2 Relative to Baseline in Children 2 Years After MIS-C Hospitalization and Age-Matched Controls

This supplemental material has been provided by the authors to give readers additional information about their work.

© 2025 Rollins. *JAMA Network Open*

eMethods. Supplemental Methods

Activity and sleep data were tracked for up to 10 days using a wearable device (FitBit Inspire 2). For data to be included in analyses, participants were required to have at least 5 calendar days of data, each meeting minimum criteria of 3 hours for sleep and 10 hours for activity, with these two variables considered separately (i.e., a patient could have activity data included but not sleep, if minimum threshold was met for activity but not sleep).The activity threshold was chosen based on existing literature.[1,2] As there are no universally established guidelines for sleep wear-time thresholds, this criterion ensures accurate tracking of sleep stages and detailed sleep metrics. Fitbit's algorithm requires a minimum of 3 hours of sleep time to provide reliable sleep data, and many sleep-related metrics depend on this duration being recorded (e.g. sleep staging). Average steps and average total sleep were recorded based on summary statistics provided by FitBit using their algorithm (based on heart rate and accelerometer) which has been shown to be valid.[3-5] Participants were initially requested to wear the watch for 1 week, but due to challenges in obtaining 5 consecutive days, the protocol duration was increased to request up to 10 days of wear. Participants were instructed to maintain a diary which used to validate the days worn.

© 2025 Rollins. *JAMA Network Open*

eTable 1. Characteristics of MIS-C Cases Returning vs non-Returning at Year 2

Variable	Participants, No. (%)			
	Returning MIS-C Cases (n = 59)	Lost MIS-C Cases (n = 5)	Returning Controls (n = 34)	Lost Controls (n = 10)
Demographic and Medical Characteristics				
Age at year 1, median (IQR), y	10.0 (9.0 to 14.0)	13.0 (11.0 to 14.0)	11.0 (10.3 to 14.8)	15.0 (10.5 to 16.0)
Sex, n (%)				
Female	19 (32)	1 (20)	17 (50)	3 (30)
Male	40 (68)	4 (80)	17 (50)	7 (70)
Race, n (%)				
Black/African American	12 (20)	1 (20)	3 (9)	2 (20)
Multiracial	5 (8)	0	4 (12)	0
White	35 (59)	4 (80)	22 (65)	6 (60)
Other or unknown	7 (12)	0	5 (15)	2 (20)
Hispanic ethnicity, n (%)	9 (15)	1 (20)	3 (9)	2 (20)
Social Vulnerability Index, median (IQR)	0.36 (0.20 to 0.60)	0.49 (0.43 to 0.50)	0.30 (0.10 to 0.54)	0.51 (0.49 to 0.94)
Primary caregiver education, n (%)				
Graduated college or greater	37 (63)[a]	1 (20)	24 (71)	4 (40)
Some college	14 (24)[a]	4 (80)	6 (18)	4 (40)
Grade 12 or less	8 (14)[a]	0	4 (11)	2 (20)
Insurance, n (%)				
Private insurance	42 (71)	4 (80)	28 (82)	7 (70)
Government insurance	16 (27)	0	6 (18)	1 (10)
Unknown	1 (2)	1 (20)	0	2 (20)
Single family home, n (%)	49 (83)	5 (100)	30 (83)	9 (90)
Baseline medical conditions, n (%)	16 (27)	3 (60)	5 (15)	2 (20)
Psychological and Medical Outcomes at Year 1, mean (SD)				
Wechsler Intelligence Scales (WASI-II, WISC-V, WAIS-IV)				
IQ estimate	101.3 (14.6)	96.2 (15.8)	104.3 (13.6)	101.0 (16.6)
Working Memory Index	99.4 (15.0)	95.4 (16.8)	103.6 (13.5)	100.6 (23.1)
Processing Speed Index	98.5 (15.9)	90.2 (15.9)	97.6 (14.4)	93.9 (14.6)
National Institutes of Health Cognition Toolbox				
List Sort Working Memory	96.3 (14.0)	94.2 (19.2)	104.2 (10.5)	97.7 (9.1)
Picture Sequence Memory	99.6 (15.4)	92.0 (8.2)	103.8 (17.7)	98.6 (20.1)
Delis-Kaplan Executive Function				
Verbal Fluency Switching	10.9 (3.1)	8.5 (2.1)	11.0 (2.7)	9.4 (1.8)
Color-Word Interference Switching	9.0 (3.3)	9.8 (1.7)	9.7 (2.9)	9.9 (4.5)

© 2025 Rollins. *JAMA Network Open*

Variable	Participants, No. (%) Returning MIS-C Cases (n = 59)	Lost MIS-C Cases (n = 5)	Returning Controls (n = 34)	Lost Controls (n = 10)
BRIEF Global Executive Composite[b]	51.8 (11.3)	61.8 (11.3)	49.8 (10.2)	54.0 (12.6)
Behavior Assessment Scale for Children, Third Edition				
Internalizing problems	52.7 (13.4)	63.2 (18.0)	47.5 (8.5)	49.3 (5.3)
Externalizing problems	49.7 (11.1)	54.0 (10.0)	47.2 (8.8)	46.5 (4.1)
Behavioral Symptoms Index[c]	50.1 (12.2)	58.2 (12.4)	47.6 (10.6)	45.7 (4.8)
PedsQL child self-report total score	80.1 (13.1)	71.0 (10.6)	85.9 (12.8)	83.5 (10.7)
PedsQL parent proxy-report total score	80.6 (15.2)	74.3 (23.6)	89.5 (12.0)	83.9 (17.8)
PROMIS Sleep Disturbance	53.7 (9.7)	51.7 (11.8)	50.4 (7.9)	52.4 (7.5)
PROMIS Sleep Impairment	49.1 (9.5)	57.1 (12.1)	47.3 (9.4)	50.5 (10.1)
Other Medical Characteristics				
Length of stay (continuous), median (IQR)	5.0 (4.0 to 8.0)	8.0 (5.0 to 9.0)	NA	NA
Length of stay (> 7 days), n (%)	16 (27)	2 (40)	NA	NA
ICU stay, n (%)	31 (53)	3 (60)	NA	NA
ICU length of stay, median (IQR)	2.0 (1.0 to 3.5)	2.0 (1.5 to 4.0)	NA	NA
Need for CPR or shock, n (%)	12 (20)[a]	4 (80)	NA	NA
Highest CRP, median (IQR)	18.8 (11.7 to 24.3)	21.0 (15.2 to 32.1)	NA	NA
Steroid exposure, n (%)	49 (83)	5 (100)	NA	NA
Left ventricular ejection fraction, n (%)				
Normal	37 (65)	1 (20)	NA	NA
Mild	11 (19)	3 (60)	NA	NA
Moderate	6 (11)	1 (20)	NA	NA
Severe	3 (5)	0	NA	NA
Neurologic symptoms at presentation, n (%)	10 (17)	2 (40)	NA	NA

Abbreviations: BRIEF, Behavior Rating Inventory of Executive Function; CPR, cardiopulmonary resuscitation; CRP, C-reactive protein (mg/dL); MIS-C, multisystem inflammatory syndrome in children; NA, not applicable; PedsQL, Pediatric Quality of Life Inventory; PROMIS, Patient Reported Outcomes Measurement Information System; WAIS-IV, Wechsler Adult Intelligence Scale, Fourth Edition; WASI-II, Wechsler Abbreviated Scale of Intelligence, Second Edition; WISC-V, Wechsler Intelligence Scale for Children, Fifth Edition.

[a] Returning vs lost groups are significantly different (P-value < 0.05).

[b] Includes BRIEF-2 and BRIEF-A.

[c] Includes depression, hyperactivity, aggression, atypicality, withdrawal, and attention.

© 2025 Rollins. *JAMA Network Open*

eTable 2. Services Received by MIS-C Cases and Controls Between Year 1 and Year 2

Rehabilitative Service	Participants, No. (%)	
	MIS-C cases (n = 58)	Controls (n = 36)
Occupational therapy	1 (2)	1 (3)
Speech therapy	4 (7)	1 (3)
Physical therapy	6 (10)	2 (6)
Behavioral or cognitive therapy	19 (33)	7 (19)

© 2025 Rollins. *JAMA Network Open*

eTable 3. General Impressions of Health Status at Year 2 Relative to Baseline in Children 2 Years After MIS-C Hospitalization and Age-Matched Controls

Variable	Participants, No. (%)		
	MIS-C Cases (n = 58)	Controls (n = 36)	Risk Difference (95% CI)[a]
Energy			
Worse than baseline (< 100% of baseline)	15/58 (26)	4/36 (11)	15 (1 to 29)[b]
< 90% of baseline	7 (12)	3 (8)	4 (-8 to 15)
< 75% of baseline	3 (5)	2 (6)	0 (-10 to 9)
Appetite			
Worse than baseline	9/58 (16)	0/36	16 (6 to 25)[c]
< 90% of baseline	5 (9)	0	-
< 75% of baseline	1 (2)	0	-
Sleep			
Worse than baseline	19/58 (33)	2/35 (6)	27 (15 to 39)[c]
< 90% of baseline	13 (22)	2 (6)	17 (6 to 27)[c]
< 75% of baseline	4 (7)	1 (3)	4 (-2 to 10)
Cognition			
Worse than baseline	17/58 (29)	2/35 (6)	24 (12 to 35)[c]
< 90% of baseline	7 (12)	2 (6)	6 (-2 to 14)
< 75% of baseline	2 (3)	1 (3)	1 (-3 to 5)
Mood			
Worse than baseline	16/58 (28)	5/36 (14)	14 (0 to 28)
< 90% of baseline	10 (17)	5 (14)	3 (-9 to 15)
< 75% of baseline	4 (7)	4 (11)	-4 (-15 to 7)

[a] Risk difference and 95% confidence intervals are calculated from a binary regression model using the identity link with generalized estimating equations and the independence working assumption to accommodate matched pairs.
[b] MIS-C cases and controls are significantly different (P-value < 0.05).
[c] MIS-C cases and controls are significantly different (P-value < 0.01).

© 2025 Rollins. *JAMA Network Open*

eFigure 1. Parallel Line Plot Depicting Group Differences and 2-Year Change in National Institute of Health Cognition Toolbox List Sort Working Memory

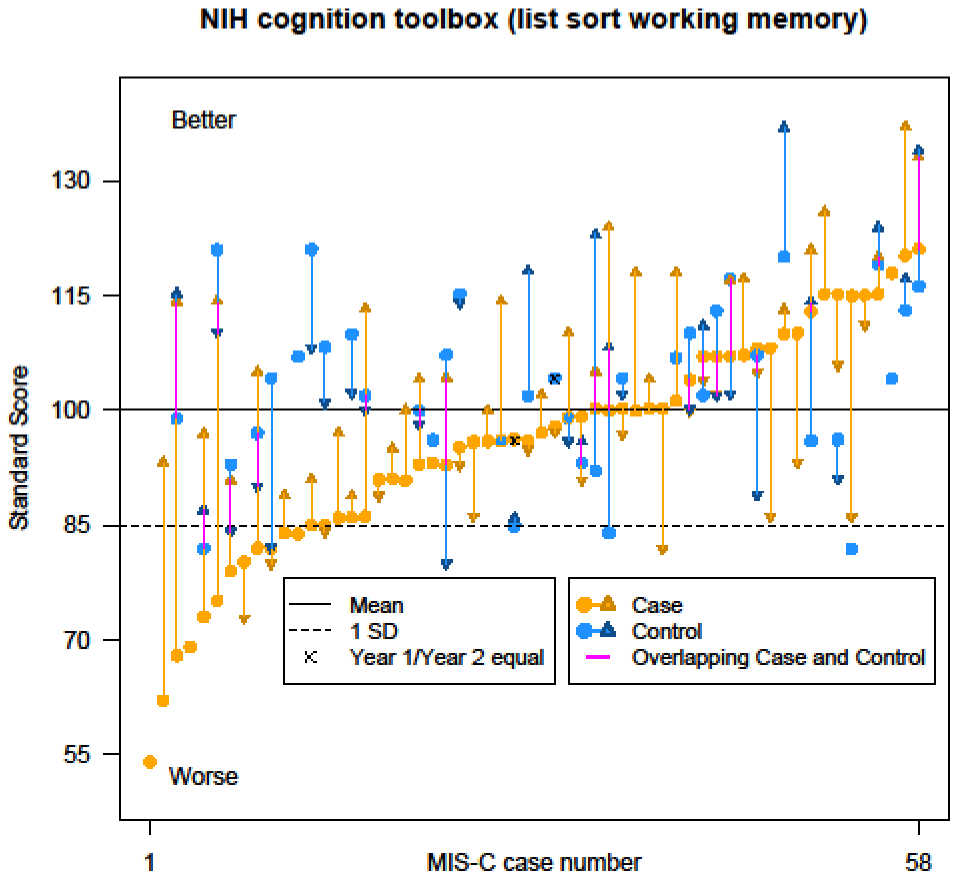

Legend: 58 patients with multisystem inflammatory syndrome in children (MIS-C) are plotted in orange, 38 controls in blue, sorted from lowest to highest Year 1 score. Lines and arrow reflect change between Year 1 and Year 2 score, where available. Matched controls, where available, are plotted on the same x-axis value as the case. Purple lines signify overlapping values between cases and their matched control. Solid black line reflects test mean. Dotted black line indicates 1 SD worse than the mean.

© 2025 Rollins. *JAMA Network Open*

eFigure 2. Parallel Line Plot Depicting Group Differences and 2-Year Change in PedsQL Generic Self-Report Total Score

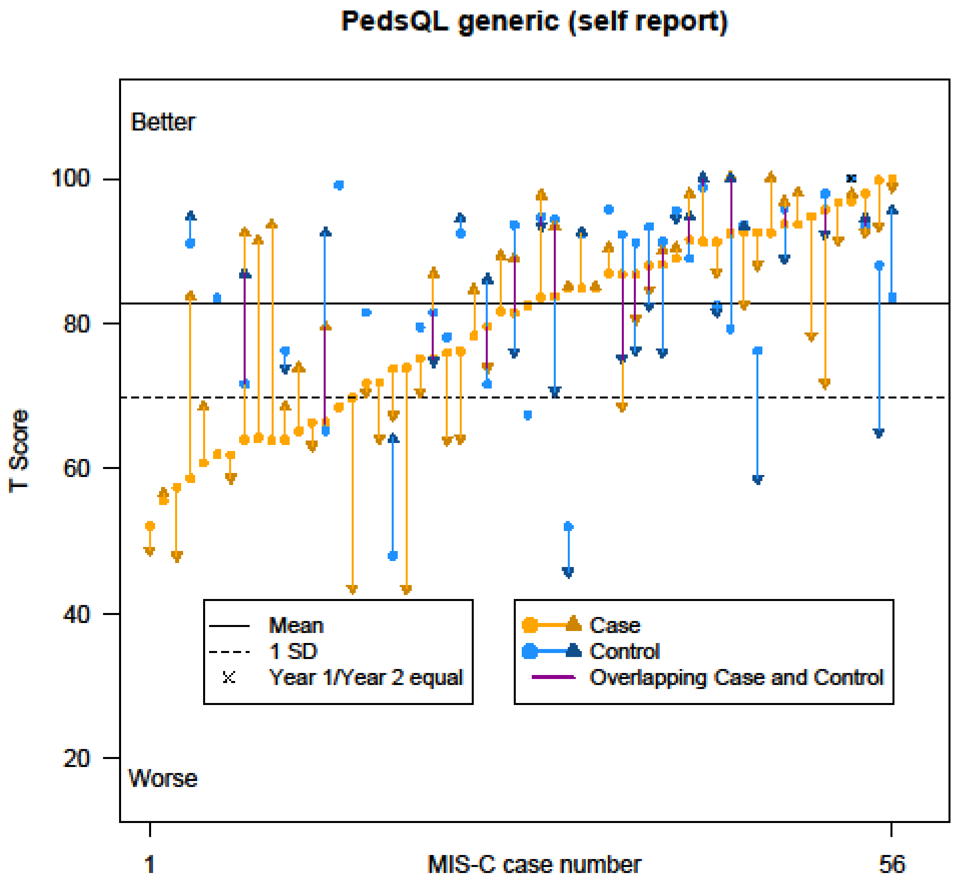

Legend: 56 patients with multisystem inflammatory syndrome in children (MIS-C) are plotted in orange, 37 controls in blue, sorted from lowest to highest Year 1 score. Lines and arrow reflect change between Year 1 and Year 2 score, where available. Matched controls, where available, are plotted on the same x-axis value as the case. Purple lines signify overlapping values between cases and their matched control. Solid black line reflects test mean. Dotted black line indicates 1 SD worse than the mean.

© 2025 Rollins. *JAMA Network Open*

eFigure 3. Parallel Line Plot Depicting Group Differences and 2-Year Change in PedsQL Generic Parent-Report Total Score

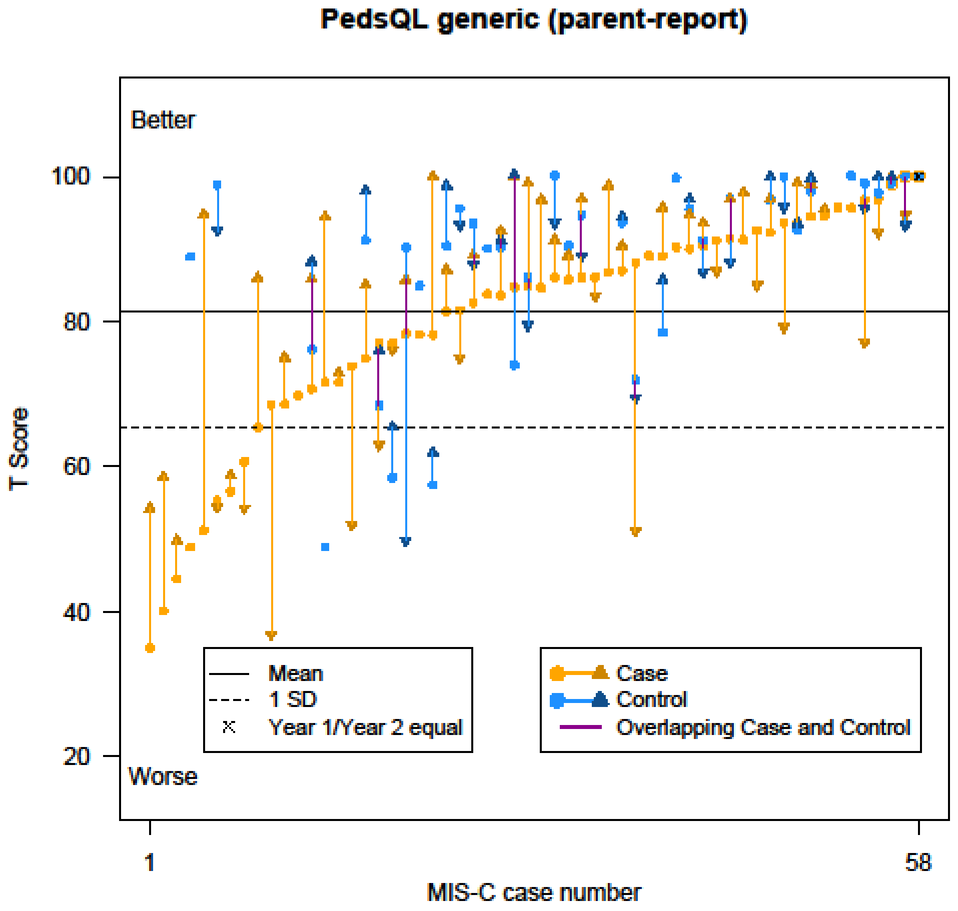

Legend: 58 patients with multisystem inflammatory syndrome in children (MIS-C) are plotted in orange, 37 controls in blue, sorted from lowest to highest Year 1 score. Lines and arrow reflect change between Year 1 and Year 2 score, where available. Matched controls, where available, are plotted on the same x-axis value as the case. Purple lines signify overlapping values between cases and their matched control. Solid black line reflects test mean. Dotted black line indicates 1 SD worse than the mean.

© 2025 Rollins. *JAMA Network Open*

References

1. Tudor-Locke C, Camhi SM, Troiano RP. A catalog of rules, variables, and definitions applied to accelerometer data in the National Health and Nutrition Examination Survey, 2003-2006. *Prev Chronic Dis*. 2012;9:E113. PMC3457743
2. Troiano RP, Berrigan D, Dodd KW, Mâsse LC, Tilert T, McDowell M. Physical activity in the United States measured by accelerometer. *Med Sci Sports Exerc*. 2008;40(1):181-8.
3. Burkart S, Beets MW, Armstrong B, et al. Comparison of multichannel and single-channel wrist-based devices with polysomnography to measure sleep in children and adolescents. *J Clin Sleep Med*. 2021;17(4):645-652. PMC8020711
4. Bagot KS, Matthews SA, Mason M, et al. Current, future and potential use of mobile and wearable technologies and social media data in the ABCD study to increase understanding of contributors to child health. *Dev Cogn Neurosci*. 2018;32:121-129. PMC6447367
5. Menghini L, Yuksel D, Goldstone A, Baker FC, de Zambotti M. Performance of Fitbit Charge 3 against polysomnography in measuring sleep in adolescent boys and girls. *Chronobiol Int*. 2021;38(7):1010-1022. PMC8255273

© 2025 Rollins. *JAMA Network Open*
