# Supplementary material for: Neurologic and Psychological Outcomes 2 Years After Multisystem Inflammatory Syndrome in Children
Source: JAMA Netw Open. 2025 Jun 2;8(6):e2512487. doi: 10.1001/jamanetworkopen.2025.12487 (PMC12131094; doi:10.1001/jamanetworkopen.2025.12487)
Supplement: Supplement 2. — Nonauthor Collaborators. The Overcoming COVID-19 Investigators [file jamanetwopen-e2512487-s002.pdf]

Supplemental Online Content: Nonauthor Collaborators
*First name, last name, and suffix (if applicable) are required and will appear in PubMed.

*Group Name(s): Overcoming COVID-19 Investigators									
***First Name and Middle Initial(s)**	***Last Name**	***Suffix (eg, Jr, III)**	Academic Degrees	Institution	Location (city, state/province, country)	Role or Contribution, eg, chair, principal investigator	Group (if more than 1 Group listed in the byline) and/or Subgroup (eg, Steering Committee)		
Meghan	Murdock		RN	University of Alabama at Birmingham	Birmingham, AL	Clinical Research Coordinator	Overcoming COVID-19 Investigators		
Heather	Kelly		RN	University of Alabama at Birmingham	Birmingham, AL	Clinical Research Coordinator	Overcoming COVID-19 Investigators		
Candice	Colston		associate degree	University of Alabama at Birmingham	Birmingham, AL	Clinical Research Coordinator	Overcoming COVID-19 Investigators		
Margaret	Newhams		MPH	Boston Children's Hospital	Boston, MA	Project Manager	Overcoming COVID-19 Investigators		
Tina	Pouissaint		MD	Boston Children's Hospital	Boston, MA	Coinvestigator	Overcoming COVID-19 Investigators		
Lora Martin	Martin		MSN, APRN, FNP-C	University of Mississippi Medical Center	Jackson, MS	Researcher	Overcoming COVID-19 Investigators		
Lacy	Malloch		BSc	University of Mississippi Medical Center	Jackson, MS	Coordinator/Project Manager	Overcoming COVID-19 Investigators		
Ashley	Stanley-Copeland		MD	University of Mississippi Medical Center	Jackson, MS	Data collection	Overcoming COVID-19 Investigators		
Jeanie	Craft		None	University of Mississippi Medical Center	Jackson, MS	Coordinator	Overcoming COVID-19 Investigators		
