# Supplementary material for: Neurologic and Psychological Outcomes 2 Years After Multisystem Inflammatory Syndrome in Children
Source: JAMA Netw Open. 2025 Jun 2;8(6):e2512487. doi: 10.1001/jamanetworkopen.2025.12487 (PMC12131094; doi:10.1001/jamanetworkopen.2025.12487)
Supplement: Supplement 3. — Data Sharing Statement [file jamanetwopen-e2512487-s003.pdf]

Data Sharing Statement

Rollins. Neurologic and Psychological Outcomes 2 Years After Multisystem Inflammatory Syndrome in Children. *JAMA Netw Open*. Published June 02, 2025.
doi:10.1001/jamanetworkopen.2025.12487

Data
Data available: Yes
Data types: Deidentified participant data
How to access data: Upon request to the corresponding author at
caitlin.rollins@childrens.harvard.edu
When available: With publication

Supporting Documents
Document types: None

Additional Information
Who can access the data: Researchers whose proposed use of the data has been approved.
Types of analyses: Analyses for which the data has a reasonable specified purpose.
Mechanisms of data availability: Following proposal approval and with a signed data access agreement.
